# Incidence and risk factors for poor ankle functional recovery, and the development and progression of posttraumatic ankle osteoarthritis after significant ankle ligament injury (SALI): the SALI cohort study protocol

**DOI:** 10.1186/s12891-021-04230-8

**Published:** 2021-04-17

**Authors:** Thomas Bestwick-Stevenson, Laura A. Wyatt, Debbie Palmer, Angela Ching, Robert Kerslake, Frank Coffey, Mark E. Batt, Brigitte E. Scammell

**Affiliations:** 1 Academic Orthopaedics, Trauma and Sports Medicine, School of Medicine, University of Nottingham, Nottingham, UK; 2 The Centre for Sport, Exercise and Osteoarthritis Research Versus Arthritis, Queen’s Medical Centre, Nottingham, UK; 3 Institute of Sport, PE and Health Sciences, Moray House School of Education and Sport, University of Edinburgh, Edinburgh, UK; 4 Centre for Health Sciences Research, School of Health and Society, University of Salford, Salford, Greater Manchester, UK; 5 Nottingham University Hospital NHS Trust, Nottingham, UK

**Keywords:** Ankle sprain, Osteoarthritis, Injury, Cohort study, Study protocol

## Abstract

**Background:**

Ankle sprains are one of the most common musculoskeletal injuries, accounting for up to 5% of all Emergency Department visits in the United Kingdom. Ankle injury may be associated with future ankle osteoarthritis. Up to 70% of ankle osteoarthritis cases may be associated with previous ankle injury. There is limited research regarding the association between ankle sprain and ankle osteoarthritis development. The current study aims to phenotype those who suffer significant ankle ligament injuries, identify potential risk factors for ankle injuries and subsequent poor recovery, examine why individuals may develop osteoarthritis, and what factors influence this chance.

**Methods:**

In this multicentre cohort study participants were recruited from nine Emergency Departments and two Urgent Care Centres in the United Kingdom. Participants (aged 18–70 years old) were defined as those who had suffered an isolated acute ankle sprain, which was Ottawa Ankle Rules positive, but negative for a significant ankle fracture on x-ray. Age and sex matched controls were also recruited. The controls were individuals who had not suffered a significant ankle injury, including ankle pain, function affected for more than 7 days, or the ankle caused them to report to an Emergency Department. Data is collected through a series of seven questionnaires (at baseline, 3 months, 1 year, 3 years, 5 years, 10 years, and 15 years later). The questionnaires include four sections (demographic questions; index injury, and injury history questions; functional assessment questions; and quality of life questions) and are designed to collect detailed information about the individual, their injury, potential risk factors for ankle sprains and ankle osteoarthritis, plus their medical history and any medication consumed.

**Discussion:**

The Significant Ankle Ligament Injury (SALI) study aims to add to the limited knowledge regarding which factors can predict ankle sprains, complaints, and osteoarthritis. This is important because despite ankle sprains being regarded as a benign injury that resolves quickly, residual symptoms are not uncommon months and years after the injury.

**Supplementary Information:**

The online version contains supplementary material available at 10.1186/s12891-021-04230-8.

## Background

Ankle sprains are one of the most common musculoskeletal injuries [1–7]. This is emphasised by research, worldwide, stating the high prevalence of ankle sprains [8–12]. Ankle sprains account for up to 5% of all Emergency Department visits in the UK annually, which is approximately 5600 injuries per day [4, 8, 13–16].

Ankle sprains are also the most common type of ankle injury in many sports [14, 17–21], with about half of all reported ankle sprain injuries occurring during sports [12, 22, 23]. This has been stated to be about 15% of the total number of sporting injuries [9] and emphasises that ankle sprains are one of the most common injuries to occur during sport and physical activity, both recreationally and competitively [20, 24–27]. This is particularly evident in sports where participants frequently jump and land on 1 foot, make sharp cutting manoeuvres, or make contact with either a teammate or opponent, such as basketball [28, 29], volleyball [30], field hockey [31], handball [32], football [33], and figure skating [34] where ankle sprains have been reported as a predominant injury. Importantly the risk of sustaining an ankle sprain is different for differing sports [2, 17, 19].

Despite the high prevalence of ankle sprains, they are often regarded as a benign and self-limiting injury, which will resolve quickly with limited treatment and respond well to conservative management [8, 14, 28, 35–37]. However, studies have shown that residual symptoms are not uncommon months and even years after the injury [14, 28, 38, 39].

Approximately, one third of patients report residual complaints after treatment, such as pain, re-sprain, swelling, muscular weakness, plus loss of function or feelings of instability [2, 6, 9, 16, 19, 22, 23, 40–42]. In studies where patients visited their GP after lateral ankle sprain, 47.5% of the patients experienced persistent complaints and only 17.5% regarded themselves as completely recovered [9, 11, 43]. Around 33% of patients experience pain 1 year after spraining an ankle [22, 42], and 50% of patients who suffer an ankle sprain experience pain 4 years after suffering the sprain [44]. Similarly, studies with a long-term follow-up, ranging from 5 to 7 years, reported chronic complaints in 20–39% of the patients [22, 45, 46]. In addition to persistent complaints, individuals also have an increased risk of recurrent ankle sprain [9, 41, 47], for example Kreitner, et al. [48] found that up to 34% of patients report at least one re-sprain within 3 years of the initial sprain.

The residual complaints have been linked to both functional and mechanical impairments [49, 50]. These impairments may contribute to long-term restrictions and limitations in occupational and recreational activities, which can produce an increased likelihood of individual’s physical activity decreasing [51, 52], and consequently negatively affects health related quality of life [46, 49, 53]. Consequently, ankle sprain injuries could represent a significant public health problem and a major health care burden [46, 54]. All of which could be due to injuries from sport, exercise, and recreation, such as an ankle sprain, being a leading cause of cessation of regular physical activity [55–57]. Thus, due to ankle sprains being a common injury [18, 56], with a high percentage of disability occurring after the initial injury, it is an injury which may play a significant role in reducing an individual’s physical activity [46, 56].

The occurrence of ankle sprains does not only have a physical impact for patients but also impacts society, through high social economic costs, and financial burden [8, 22, 41]. Both of which are associated with diagnosis, treatment, and loss of work productivity contingent with the severity of the injury [1, 3, 8]. This is shown by a quarter of individuals who sustain ankle sprain injuries being unable to attend work or school for 7 days following the initial injury [4, 8, 58]. The financial impact is also emphasised by the medical cost of one ankle sprain, including the healthcare and time off work, costing in the range of £940 to £1314, which is £1–2 billion annually [59, 60]. However, this figure only accounts for individuals who have sought help from a hospital emergency department and not those who have self-managed or received other forms of care [59, 60].

Ankle sprains have also been stated as a leading cause of post-traumatic ankle osteoarthritis [2, 8, 61–64]. Osteoarthritis is the most common form of arthritis and one of the leading causes of pain and disability [14, 65–74]. Most research has been conducted on the hip and knee, where post-traumatic osteoarthritis accounts for 8 and 12% of cases, respectively [75, 76]. In contrast up to 70% of ankle osteoarthritis cases may be associated with previous ankle injuries [76], however ankle osteoarthritis is poorly understood. The cause of osteoarthritis remains somewhat unknown, but a number of risk factors (mainly for knee and hip osteoarthritis) have been identified, including joint injury, muscle weakness, mechanical stress or loading of the joints [77–80]. It is possible that these risk factors play a role in ankle osteoarthritis however the degree to which is still unknown.

Sex and hormonal factors have also been identified as risk factors for osteoarthritis development, and so alongside a higher rate of ankle sprains [8, 81, 82] females have been found to be more susceptible than males in developing ankle osteoarthritis [77, 79, 80]. Females susceptibility to both ankle sprains and ankle osteoarthritis could be due to the anatomical [83–85], hormonal [84, 86, 87], and neuromuscular differences [86, 88, 89] which exists between sexes. However, these differences may not necessarily explain the differences in incidence rates of ankle sprains, and thus suggests that future research is needed to examine whether differences between sexes are related to anatomical or physiological differences, or if differences are activity specific and thus related to training behaviours [8].

Age has also been identified as a risk factor for both ankle sprains and osteoarthritis, with the incidence of ankle sprains being found to be higher in younger people [8, 10, 43], while the prevalence of osteoarthritis increases with age [90, 91]. Osteoarthritis is not a normal part of the ageing process. Association may be a consequence of collective exposure to various risk factors, physiological changes, and vulnerabilities which occur as part of aging, thus making the joint susceptible to osteoarthritis [69, 92]. This can include a loss of strength, increased reaction time, poor proprioception, oxidative damage, and cartilage thinning [69, 92].

There is limited longitudinal research regarding ankle osteoarthritis in general, and in particular after trauma [14, 41]. Additionally, factors which can predict persistent ankle complaints are relatively unknown [22], all of which highlights the need for further research to examine these factors. The following is a methodological description of the significant ankle ligament injury (SALI) cohort study, which aims to phenotype those who suffer significant ankle ligament injuries, identify potential risk factors for ankle injuries, examine why individuals may develop osteoarthritis, and what factors influence this chance.

### Objectives

1. To identify the prevalence of persistent ankle pain and dysfunction at discrete timepoints following presentation to the Emergency Department for a significant ankle ligament injury.
2. To determine the individual risk factors for poor patient reported outcomes measures (e.g. pain and persistent dysfunction) following significant ankle ligament injury.
3. To identify the prevalence and risk factors for development of ankle osteoarthritis, post significant ankle ligament injury.

### Methodology

**Fig. 1:**
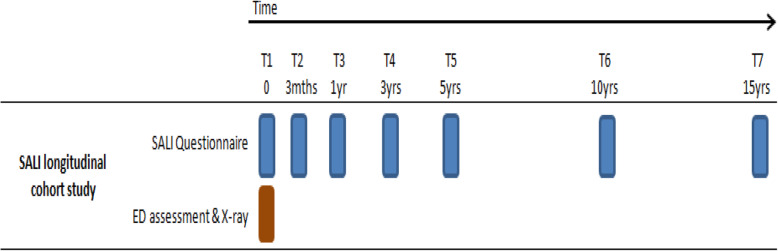
A schematic of the time points which SALI participants are followed up with questionnaires. Abbreviations: SALI; Significant Ankle Ligament Injury, ED: Emergency Department

This study is a longitudinal prospective cohort study that obtains questionnaire data from adults aged 18 to 70 who suffer a significant ankle ligament injury. Participants are recruited following attendance at an Emergency Department for their ankle injury. The study collects data at seven time points; at time of the injury, then 3 months, 1 year, 3 years, 5 years, 10 years, and 15 years after injury (Fig. 1). Further nested studies will be considered dependant on ethical review and funding.

### Ethics

All aspects of this study were approved by the Nottingham Research Ethics Committee (NREC reference: 15/EM/0384), and the Research & Innovation department of Nottingham University Hospitals NHS Trust (study sponsor).

### Eligibility criteria

#### Inclusion

Eligible patients were aged between 18 and 70 years old at the time of seeking medical attention for an isolated acute (within 2 weeks) ankle sprain. Patients were also required to be Ottawa Ankle Rules positive, but negative for a significant ankle fracture on an x-ray. Ottawa Ankle Rules positive is when an individual reports pain in the malleolar zone and any of the following; bone tenderness at the posterior edge (6 cm) or tip of the lateral malleolus; or bone tenderness at the posterior edge (6 cm) or tip of the medial malleolus; or an inability to bear weight immediately and in the Emergency Department for four steps [7, 93]. Individuals who suffered a flake (avulsion) fracture were also included, due to flake fractures being able to be treated as soft-tissue injuries, as reported in other studies [15, 16]. Eligibility was assessed by the clinical team in each Emergency Department, or the study team following thorough training.

#### Exclusion

Patients aged under 18 and over 70 were excluded, in addition to those with injuries 2 weeks old or greater and significant ankle fracture (with the exception of flake fractures [4, 15, 16]), as reported by x-ray [94]. Patients with other significant joint injuries, including additional lower leg injury, and bilateral ankle injury, were excluded [6, 16]. Individuals were also excluded if they were not independently living or could not read English, like the study Matthews, et al. [95] conducted.

### Recruitment

This multi-centre study recruited participants from nine Emergency Departments and two Urgent Care Centres in the United Kingdom. The recruitment sites were; Nottingham University Hospital Queens Medical Centre, Leeds General Infirmary, Leeds St James University Hospital, Royal Infirmary of Edinburgh, University Hospital Coventry, Royal Berkshire Hospital, Warwick Hospital, King’s Mill Hospital, and Royal Derby Hospital. In addition to two Urgent Care Centres; Nottingham Urgent Care Centre and St Cross, Rugby. Eligible patients were identified and first approached by Emergency Department clinical staff in one of two ways:

### Phase one

Face-to-face contact by Emergency Department clinicians (Emergency Nurse Practitioners, Advanced Nurse Practitioners, Initial Assessment Team, Emergency Department Physiotherapists, or doctors).

### Phase two

Retrospective identification using medical records within 2 weeks of the patient’s first contact with the Emergency Department.

#### Phase one

Ankle injury patients were given an introductory SALI study Patient Information Sheet before their x-ray. If their x-ray was negative, the patient was given more information about the study while they were in the Emergency Department by the clinical team. If interested, the research team explained the study in detail, giving opportunity to ask questions. Informed consent was received from the patients. Once enrolled participants were given a unique SALI ID number, SALI cohort study covering letter, a questionnaire (baseline), and a pre-paid business envelope for them to return the questionnaire in. Alternatively, participants could also receive an email link to the questionnaire.

Interested participants that were unable to stay to speak with the research team provided their name, postal and email addresses, and contact telephone number for telephone follow-up by the research team. Their hospital identifier number was noted (NHS number or site specific) by the clinician. For patients that declined participation their hospital identifier number only was recorded (to avoid contacting these patients following retrospective phase two screening).

#### Phase two

Patients that attended the Emergency Department with an ankle injury in the previous week were retrospectively screened by the clinical team. Eligible patients (that were not approached by phase one recruitment) were posted a letter of introduction, participant information sheet, informed consent form, contact details form and prepaid return envelope. Two weeks following the initial postal contact patients was followed up by telephone, having had time to consider if they wish to take part in the study. Consenting participants were enrolled in the study and sent the baseline questionnaire, either by post, with a pre-paid reply envelope, or by email, depending on their preference.

Recruitment was also aided through the use of posters in the Emergency Departments. These posters provided brief study details, and the research team contact details.

### Control participants

Age and sex matched controls from the general population were also recruited. The control participants were any individual who had not suffered a significant ankle injury (e.g. one that caused ankle pain and poor function for more than 7 days) and caused them to report to an Emergency Department. The control participants were recruited through posters and leaflets in hospital Emergency Departments and around the University of Nottingham.

### Sample size

A sample size calculation was conducted, based on a 6 month pilot study at Queens Medical Centre, Nottingham. This recorded 2723 ankle sprain patients reporting to Emergency Department, 1760 of which were Ottawa Ankle Rules positive, but negative for a significant ankle fracture on an x-ray. Thus, we estimated ~ 7000 ankle sprain patients, which were Ottawa Ankle Rules positive, but negative for a significant ankles fracture on x-ray, would report to Queens Medical Centre Emergency Department in a 24 month period, with ~ 4600 being aged between 18 and 70, thus eligible for the study. Consequently, based on a recruitment rate of about 40%, and the Emergency Departments varying in size between the study’s recruitment sites we aim to recruit 3000 participants.

### Duration of the study

Recruitment began in October 2016 for a period of 39 months, with the last patient recruited in January 2020. The recruited participants are followed up for a period of 15 years, with questionnaires sent serially (Fig. 1). Due to the study being a 15 year cohort study the recruitment is not currently completed for all time points, thus the overall recruitment is still ongoing.

### Questionnaires

Consenting participants complete a questionnaire at each data collection time point (Fig. 1). Questionnaires differ slightly at each time point (Table 1). If a participant does not respond to a questionnaire, they are sent up to three reminders, at around 2, 4, and 6 weeks after the questionnaire was sent. Reminders consist of a telephone call, email, or postal reminder. The questionnaires include four sections; demographics; index injury and injury history; functional assessments; and quality of life (see Additional file 1). The questionnaires collect detailed information about the individual and their injury, potential risk factors for ankle sprains and ankle osteoarthritis, in addition to comorbidities and medication consumed. A pilot baseline questionnaire was completed by volunteers before the start of the study, to assess content, language and length. Patient and public involvement was coordinated through the Centre for Sport, Exercise and Osteoarthritis Research Versus Arthritis and Nottingham University Hospital NHS Trust.

**Table 1:** Data recorded at each questionnaire timepoint

		Follow up assessments					
	Initial assessment	3 months	1 year	3 years	5 years^a^	10 years^a^	15 years^a^
**SECTION ONE: BASELINE DATA**							
**Demographics**							
Date of birth	X	X	X	X	X	X	X
Sex	X						
Ethnicity	X						
Body Mass Index (height, body mass)	X	X	X	X	X	X	X
Limb dominance (hand/foot)	X						
Education history	X			X			
Marital status	X						
Occupational history	X			X	X		
Female specific (start and end of menstrual cycle)	X						
Diet and nutrition	X						
**Family joint health**							
Family history of joint replacement	X				X	X	X
Family history of osteoarthritis	X				X	X	X
**Participant joint health**							
History of joint surgery	X	X	X	X	X	X	X
Diagnoses of osteoarthritis	X	X	X	X	X	X	X
**Line drawings**							
Pain mannequin	X	X	X	X	X	X	X
Knee angle alignment	X		X	X	X	X	X
Heberden’s Nodes	X		X	X	X	X	X
2D:4D ratio	X				X	X	X
Foot position and arches	X				X	X	X
Flexibility; knee, forward bending, elbow, thumb and finger bend	X				X	X	X
Hypermobility	X				X	X	X
**SECTION TWO: ANKLE INJURY**							
**Emergency Department attended ankle injury**							
Place of injury (e.g. sport, work, home, other)	X						
Cause of injury (including footwear at time)	X						
Ankle pain score	X	X	X	X	X	X	X
Care pathway	X	X					
Injury treatment	X	X		X	X	X	X
Severity - time to return to normal function from ankle injury	X	X	X	X			
General injury history	X	X	X		X	X	X
Subsequent injury history		X	X	X	X	X	X
**SECTION THREE: ANKLE FUNCTIONAL ASSESSMENTS**							
Chronic ankle instability	X	X	X	X	X	X	X
Foot and ankle outcome score	X	X	X	X	X	X	X
**SECTION FOUR: HEALTH AND QUALITY OF LIFE**							
**Activity assessment**							
Work related activity assessment	X	X	X	X	X	X	X
Physical activity; and sport and leisure assessment	X	X	X	X	X	X	X
Tobacco and alcohol use	X	X	X	X	X	X	X
Current disease	X	X	X	X	X	X	X
Medication	X	X	X	X	X	X	X
Family disease history	X	X	X	X	X	X	X
EQ-5D-5L	X	X	X	X	X	X	X

^a^Questionnaires are currently being developed/designed. Content may change slightly

The demographic section records age, and sex, plus height and body mass (which is also used to calculate body mass index). This section also records known risk factors for osteoarthritis, namely knee alignment, Heberden’s nodes, and the relationship between index to ring finger (2D:4D ratio) [96]. Participants self-report these measures using validated line drawings [97, 98].

The injury section records information relating to the current ankle injury, such as which ankle was injured, the cause of the injury, and footwear worn at the time of injury. Participants are also asked to report their current level of ankle pain and pain at the time of the injury (between 0 and 10, where 0 = no pain and 10 = maximum pain). Previous ankle injuries and other significant injuries are also recorded.

The functional assessment questions includes the Foot and Ankle Outcome Score, which is a validated 42 item questionnaire that assesses ankle function in five sections; pain, other symptoms (e.g. swelling, stiffness, and range of motion), activities of daily living, sport and recreational activities, and foot and ankle related quality of life [99]. In the baseline questionnaire participants are asked to record their ankle function a week before their injury (to provide a ‘normal’ picture of their ankle pre-injury).

The final section explores quality of life. Questions document type, frequency and intensity of physical activity and exercise, smoking status, alcohol consumption, regular medication, as well as including an EQ-5D-5L health questionnaire [100].

### Statistical analysis

Statistical analysis will be conducted in SPSS (IBM SPSS Statistics, version 25) using descriptive statistics (mean (standard deviation)) for continuous variables, and number (percentage) for categorical variables. Variables will be considered significant if *p* values are 0.05 or below, using 95% confidence intervals.

## Discussion

We have described the protocol of a large UK cohort study aiming to phenotype those who suffer significant ankle sprains and identify potential risk factors for poor recovery. The study design also allows examination of risk factors for ankle osteoarthritis development following a significant ankle ligament injury. These are important questions as there is limited research regarding which factors are associated with suffering a significant ankle sprain (requiring x-ray), persistent complaints, and ankle osteoarthritis development [14, 22, 41]. Ankle sprains are often regarded as benign injuries which resolve quickly [8, 14, 28, 35–37], however, residual symptoms are not uncommon months and even years after the injury [14, 28, 38, 39].

Despite ankle osteoarthritis only accounting for 4% of all osteoarthritis cases, compared to 41% being the knee and 19% being the hip [14, 101], a large percentage of ankle osteoarthritis, 78%, is post-traumatic, compared to 9% of primary osteoarthritis [102], which is much higher than other joints [14, 76, 102, 103].

The current study questionnaire collects data on both age and sex, both of which have been suggested to be risk factors for both ankle sprains and osteoarthritis, with females being at higher risk for ankle sprains [8, 81, 82] and osteoarthritis [77, 79, 80], while the incident rates of ankle sprains are higher in young people [8, 10, 43], and the prevalence of osteoarthritis increases with age [90, 91]. The study also collects data on individual’s height and body mass, both of which have been identified as potential risk factors for ankle sprains [104, 105]. The questionnaires also includes questions regarding Heberden’s nodes, as a clinical sign of primary osteoarthritis, plus suggest a genetic predisposition to the development of osteoarthritis in several joints [106, 107].

We also examine the relationship between index to ring finger (2D:4D ratio) due to suggestions that when the index finger is shorter than the ring finger, “type 3”, being associated with knee osteoarthritis [98]. The relevance of the 2D:4D ratio and the risk of ankle osteoarthritis development is not yet known but will be explored in this study. The current study also records data on what footwear was worn at the time of injury, this is important as footwear influences the incidence of ankle injuries during sport [108] and non-sporting occasions [109].

The questionnaires also include questions regarding individual’s smoking status and alcohol consumption. The inclusion of questions regarding tobacco smoking is due to it being shown to be associated with increased musculoskeletal and overuse injury rates compared to non-smokers [110]. Plus, despite successfully stopping smoking, ex-smokers still maintained a higher injury risk in comparison to non-smokers [110]. Similarly, alcohol consumption has also been suggested to be a risk factor for musculoskeletal pain and injury [111, 112].

The study aims to disseminate the results of this study through publications in relevant peer-reviews journals and conference proceedings.

## Conclusions

In conclusion, the current study uses a series of questionnaires and aims to phenotype those who suffer significant ankle ligament injuries, identify risk factors for suffering a significant ankle ligament injury, plus examine why individuals may develop ankle osteoarthritis and what factors influence this chance. All of which is important as there is limited research regarding ankle sprains and ankle osteoarthritis, despite ankle sprains being a common injury.

## Supplementary Information

**Additional file 1.** SALI baseline questionnaire.

## Data Availability

Not applicable.

## Abbreviations

SALI: significant ankle ligament injury
2D:4D ratio: relationship between index to ring finger
ED: Emergency Department

## Declarations

The authors have nothing to declare.
