# Supplementary material for: Incidence and risk factors for poor ankle functional recovery, and the development and progression of posttraumatic ankle osteoarthritis after significant ankle ligament injury (SALI): the SALI cohort study protocol
Source: BMC Musculoskelet Disord. 2021 Apr 17;22:362. doi: 10.1186/s12891-021-04230-8 (PMC8052737; doi:10.1186/s12891-021-04230-8)
Supplement: Supplementary file 1 — Additional file 1. SALI baseline questionnaire. [file 12891_2021_4230_MOESM1_ESM.docx]

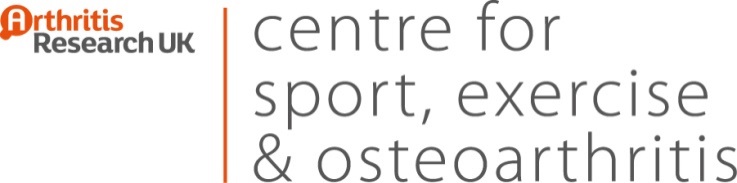

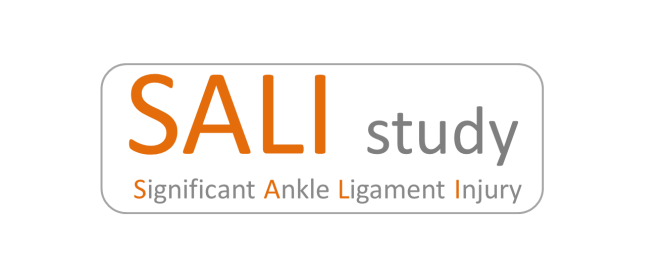


| Patient Initials |  | Date of birth |  | Unique number |  |
| --- | --- | --- | --- | --- | --- |
|  |  |  |  | SALI study ID |  |

**The Significant Ankle Ligament Injury (SALI) Cohort Study Questionnaire**

**T1 time-point**

**Version 10.3 – 10/08/16**

**Study Title**: Risk factors for the development and progression of posttraumatic ankle osteoarthritis: the SALI cohort study.

Please complete the questionnaire providing as much detail as you can. The questionnaire should take no longer than 25-30 minutes to complete.

If you have any queries about the questionnaire or any of the questions contained within it please do not hesitate to contact the research team. Dr Laura Wyatt email: laura.wyatt@nottingham.ac.uk tel. 0115 8231554.

Significant Ankle Ligament Injury (SALI) Protocol Copyright © University of Nottingham and NUH NHS trust 2015. All rights reserved. Not to be reproduced in whole or in part without the permission of the copyright owners.

| **1.1** Today’s date | / / |
| --- | --- |

**Section one: about you**

| **1.2** Date of birth | / / |
| --- | --- |

| **1.3 Gender** | Male |  | Female |  |
| --- | --- | --- | --- | --- |

**1.4** What is your **ethnicity** *(please tick the box that applies)*

| White British |  |  | Black Caribbean |  |
| --- | --- | --- | --- | --- |
| White Irish |  |  | Black African |  |
| White other |  |  | Black other |  |
| Mixed White/Black Caribbean |  |  | Asian Indian |  |
| Mixed White/Black African |  |  | Asian Pakistani |  |
| Mixed White/Asian |  |  | Asian Bangladeshi |  |
| Mixed White/other |  |  | Asian other |  |
| Chinese |  |  | Other |  |

| **1.5** What is your **height?** | | | |  | |  | |  | |  |
| --- | --- | --- | --- | --- | --- | --- | --- | --- | --- | --- |
|  | Feet |  | Inches | | **OR** | |  | | Centimetres | |

| **1.6** What is your **weight?** | | | |  | |  | |  | |  |
| --- | --- | --- | --- | --- | --- | --- | --- | --- | --- | --- |
|  | Stone |  | Ibs | | **OR** | |  | | Kilograms | |

| **1.7** Which is your **dominant hand?** | | | | | |  |
| --- | --- | --- | --- | --- | --- | --- |
| Left |  | Right |  | Ambidextrous |  | |

| **1.8** Which is your **dominant foot?** | | | | | |  |
| --- | --- | --- | --- | --- | --- | --- |
| Left |  | Right |  | Ambidextrous |  | |

**1.9** Please choose the **highest** level of **education** achieved?

| School |  |  | University graduate |  |
| --- | --- | --- | --- | --- |
| College |  |  | University post-graduate |  |

**1.10** What is your **marital status**?

| Single |  | Married |  |  | Civil partner |  |
| --- | --- | --- | --- | --- | --- | --- |
| Separated |  | Divorced |  |  | Widowed |  |

**1.11** Please provide details of the **jobs** you have had since leaving school, college or university:

| **Job title** | **Full/part time** | **Start date (dd/mm/yyyy)** | **How long for (years)** |
| --- | --- | --- | --- |
|  |  |  |  |
|  |  |  |  |
|  |  |  |  |
|  |  |  |  |
|  |  |  |  |
|  |  |  |  |
|  |  |  |  |

**1.12** Do you **currently** or have you **ever** eaten any of the diets below? If so, for how many years have you followed this diet? *(please tick all that apply)*

| Currently | Vegetarian |  | Vegan |  | Gluten free |  | No. of years |  |
| --- | --- | --- | --- | --- | --- | --- | --- | --- |
| Ever | Vegetarian |  | Vegan |  | Gluten free |  | No. of years |  |

**1.13** Do you **currently** regularly take any of the following **supplements?** *(please tick all that apply)*

| Vitamin C |  | Vitamin D |  | Vitamin E | |  | Fish oils |  | Calcium | |  | |  |  |  |
| --- | --- | --- | --- | --- | --- | --- | --- | --- | --- | --- | --- | --- | --- | --- | --- |
|  |  |  |  |  | |  |  |  |  | | |  | |  |  |
| Glucosamine |  | Chondroitin |  |  | Other supplements:___________ | | | | |  | | | | |  |

**1.14 Males:** At what age did your voice begin to change? _____

**1.15** **Females**: what age did your periods start? _____

**1.16** **Females only**; if you have **children,** at what **age** did you give birth to each of your children? *(If you have more than 4 children please include* ***your age*** *when you had your first and last child)*

| No. of children |  | Child 1 |  | Child 2 |  | Child 3 |  | Child 4 |  | Age at menopause |  |
| --- | --- | --- | --- | --- | --- | --- | --- | --- | --- | --- | --- |

**1.17** Does anyone in your **family** (any relations) have a history of **joint surgery or replacement**? *(please also indicate their age at the time)*

| **Family member** | **Body joint** | **Side (left/right)** | **Age** | **Please indicate reason for joint surgery/replacement** | | | |
| --- | --- | --- | --- | --- | --- | --- | --- |
|  |  |  |  | OA/degenerative joint |  | Accident |  |
|  |  |  |  | Rheumatoid Arthritis |  | Other |  |
|  |  |  |  | OA/degenerative joint |  | Accident |  |
|  |  |  |  | Rheumatoid Arthritis |  | Other |  |
|  |  |  |  | OA/degenerative joint |  | Accident |  |
|  |  |  |  | Rheumatoid Arthritis |  | Other |  |
|  |  |  |  | OA/degenerative joint |  | Accident |  |
|  |  |  |  | Rheumatoid Arthritis |  | Other |  |

**1.18** Please indicate any and all **members of your family** that you are aware of who have been **diagnosed with osteoarthritis (OA) or degenerative joint disease**?

| **Family member** | **Body joint** | **Side (left/right)** | **Age at the time** |
| --- | --- | --- | --- |
|  |  |  |  |
|  |  |  |  |
|  |  |  |  |
|  |  |  |  |
|  |  |  |  |
|  |  |  |  |
|  |  |  |  |
|  |  |  |  |

**
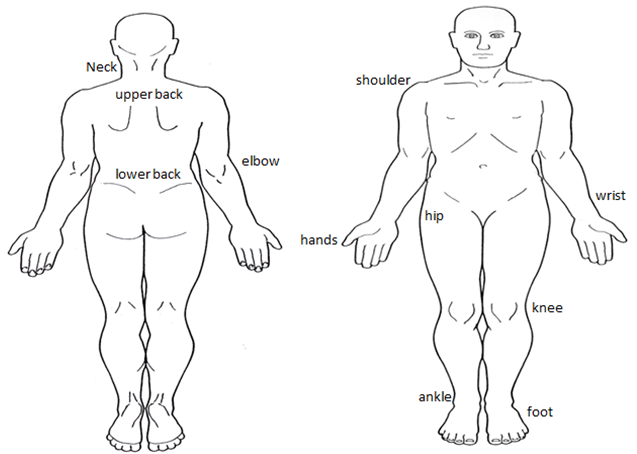
1.19** Please indicate in the space below where, by body joint, you have had **pain for most days of the prior month** AND **your level of pain** between ‘0’ no pain and ‘10’ the worst imaginable pain.

| **Body joint** | **Side of body (left/right)** | **Level of pain (0-10)** |
| --- | --- | --- |
|  |  |  |
|  |  |  |
|  |  |  |
|  |  |  |
|  |  |  |
|  |  |  |
|  |  |  |

**1.20** Have you had any **spine** or **joint surgery/replacement**? *(e.g. ankle/knee etc)*

| **Body joint** | **Side (left/right)** | **Age** | **Please indicate reason for joint surgery/replacement?** | | | |
| --- | --- | --- | --- | --- | --- | --- |
|  |  |  | OA/degenerative joint |  | Accident |  |
|  |  |  | Rheumatoid Arthritis |  | Other |  |
|  |  |  | OA/degenerative joint |  | Accident |  |
|  |  |  | Rheumatoid Arthritis |  | Other |  |
|  |  |  | OA/degenerative joint |  | Accident |  |
|  |  |  | Rheumatoid Arthritis |  | Other |  |
|  |  |  | OA/degenerative joint |  | Accident |  |
|  |  |  | Rheumatoid Arthritis |  | Other |  |

**1.21** Have **you** been **diagnosed** with **osteoarthritis** or **degenerative joint disease**?

| Yes |  | No |  |
| --- | --- | --- | --- |

**1.22** Have **you** had an **x-ray or MRI** scan to confirm **osteoarthritis**?

| Yes |  | No |  |
| --- | --- | --- | --- |

**1.23** Have **you** been told you have **osteoarthritis** by a medical professional?

| Yes |  | No |  |
| --- | --- | --- | --- |

If answered yes please complete the table below, if answered no please move to question 1.24, page 6.

| **Body joint** | **Side (left/right)** | **Age** | **Who diagnosed you?**  *(GP, Hospital doctor, Physiotherapist, Nurse, Osteopath, Chiropractor, or other)* |
| --- | --- | --- | --- |
|  |  |  |  |
|  |  |  |  |
|  |  |  |  |
|  |  |  |  |

**1.24** Please indicate which of the following pictures (below) best shows your **knee angle** alignment **currently** and in your **20s** *(if you are now aged over 30 years old)*


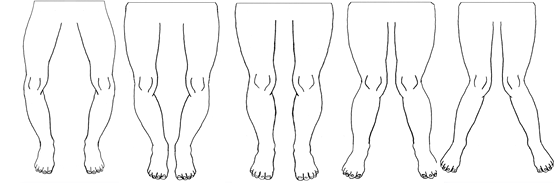


|  | Very bow legged | | | Bow legged | | | Normal | | | Knock-kneed | | | Very knock-kneed | | |
| --- | --- | --- | --- | --- | --- | --- | --- | --- | --- | --- | --- | --- | --- | --- | --- |
| Currently |  |  |  |  |  |  |  |  |  |  |  |  |  |  |  |
|  |  |  |  |  |  |  |  |  |  |  |  |  |  |  |  |
| 20s |  |  |  |  |  |  |  |  |  |  |  |  |  |  |  |

**1.25** Please indicate if you have any **finger nodes** or not (see diagram below). *A finger node is a firm, bobbly swelling on the back of the finger joint.*


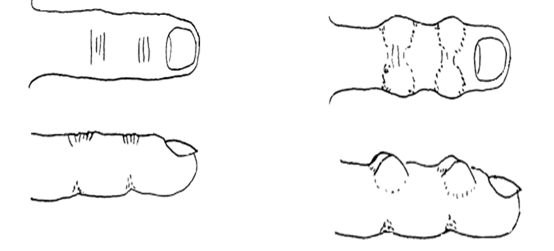


| Finger without nodes | | | Finger **with** nodes | | |
| --- | --- | --- | --- | --- | --- |
|  |  |  |  |  |  |

**1.26** Please look at **your left hand with your fingers straight in line with your forearm**. Please indicate which of the diagrams best shows the **length of your index finger (I) compared with your ring finger (R).**


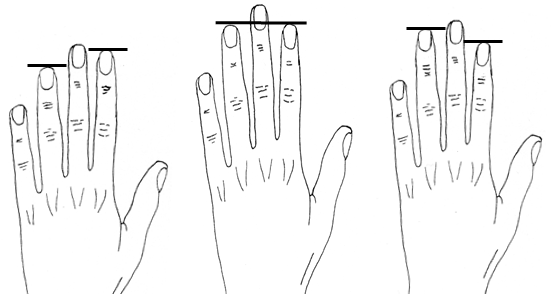


Ring finger longer than index finger

Both fingers are equal

Index finger longer than ring finger

**I**

**I**

**R**

**R**

**I**

**R**

**1.27** Please indicate which of the following **foot positions** best shows how your feet are positioned when you are stood at **rest**:


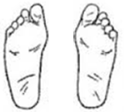

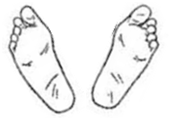

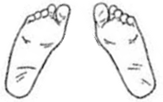


| Feet straight |  | Toes pointing out |  | Toes pointing in |  |
| --- | --- | --- | --- | --- | --- |

**1.28** Please indicate which of the following **foot arches** for each of your left and right feet best shows how your feet are normally positioned when you are stood at rest.


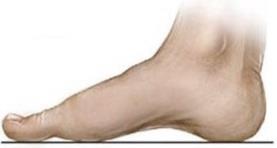

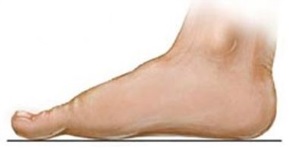

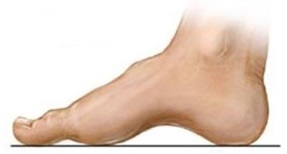


| Left foot | Normal arch |  | Flat arch |  | High arch |  |
| --- | --- | --- | --- | --- | --- | --- |
| Right foot | Normal arch |  | Flat arch |  | High arch |  |

You will be presented with a series of pictures that relate to how **flexible your joints** are. Your flexibility may differ between your right and left side so please score each limb separately where applicable. If you are over 30 years of age please also score which picture best shows your joint angles in your 20s using your best estimate.

**1.29** Which picture below best shows your how you can **bend your knee backwards?** **(please score both knees).**


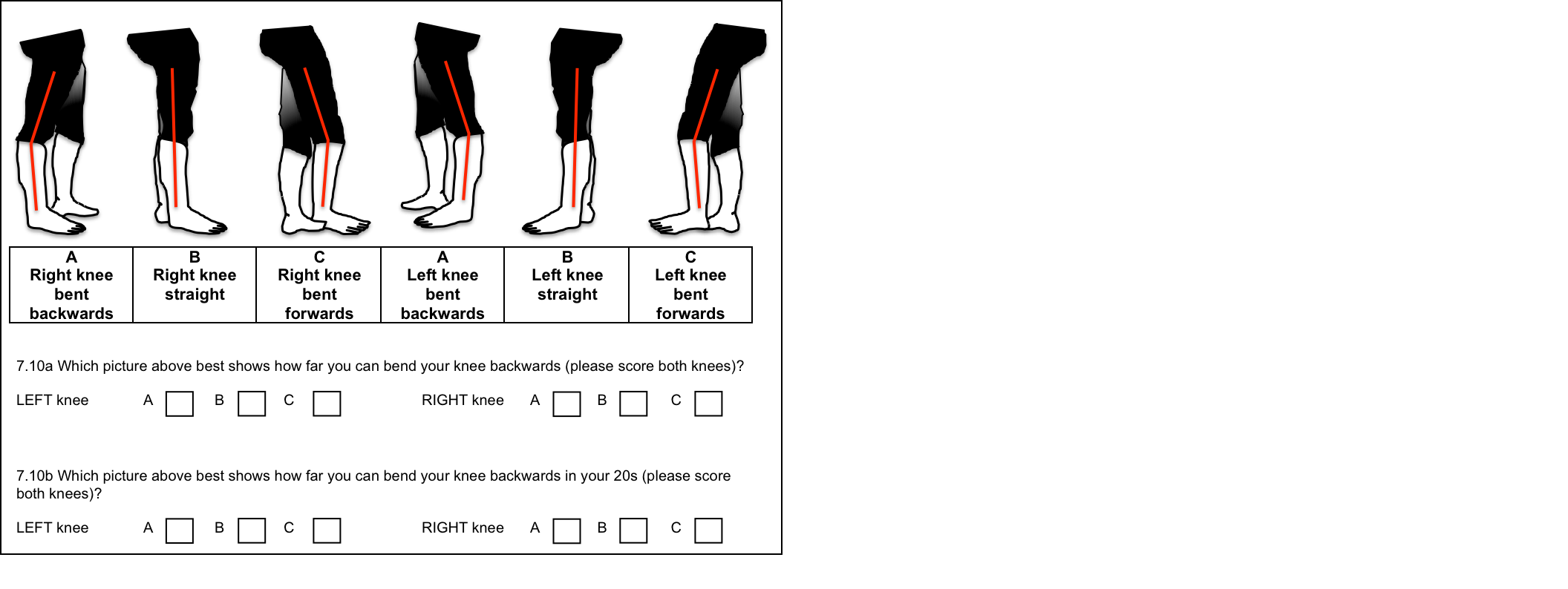


| Currently |  |  |  |  |  |  |  |  |  |  |  |  |  |  |  |  |  |
| --- | --- | --- | --- | --- | --- | --- | --- | --- | --- | --- | --- | --- | --- | --- | --- | --- | --- |
|  |  |  |  |  |  |  |  |  |  |  |  |  |  |  |  |  |  |
| 20s |  |  |  |  |  |  |  |  |  |  |  |  |  |  |  |  |  |

**1.30** Which picture describes how far you can **bend forwards without bending your knees**? Please score your joints **now**, and how you were in your **20s** (if you are over 30 years of age).


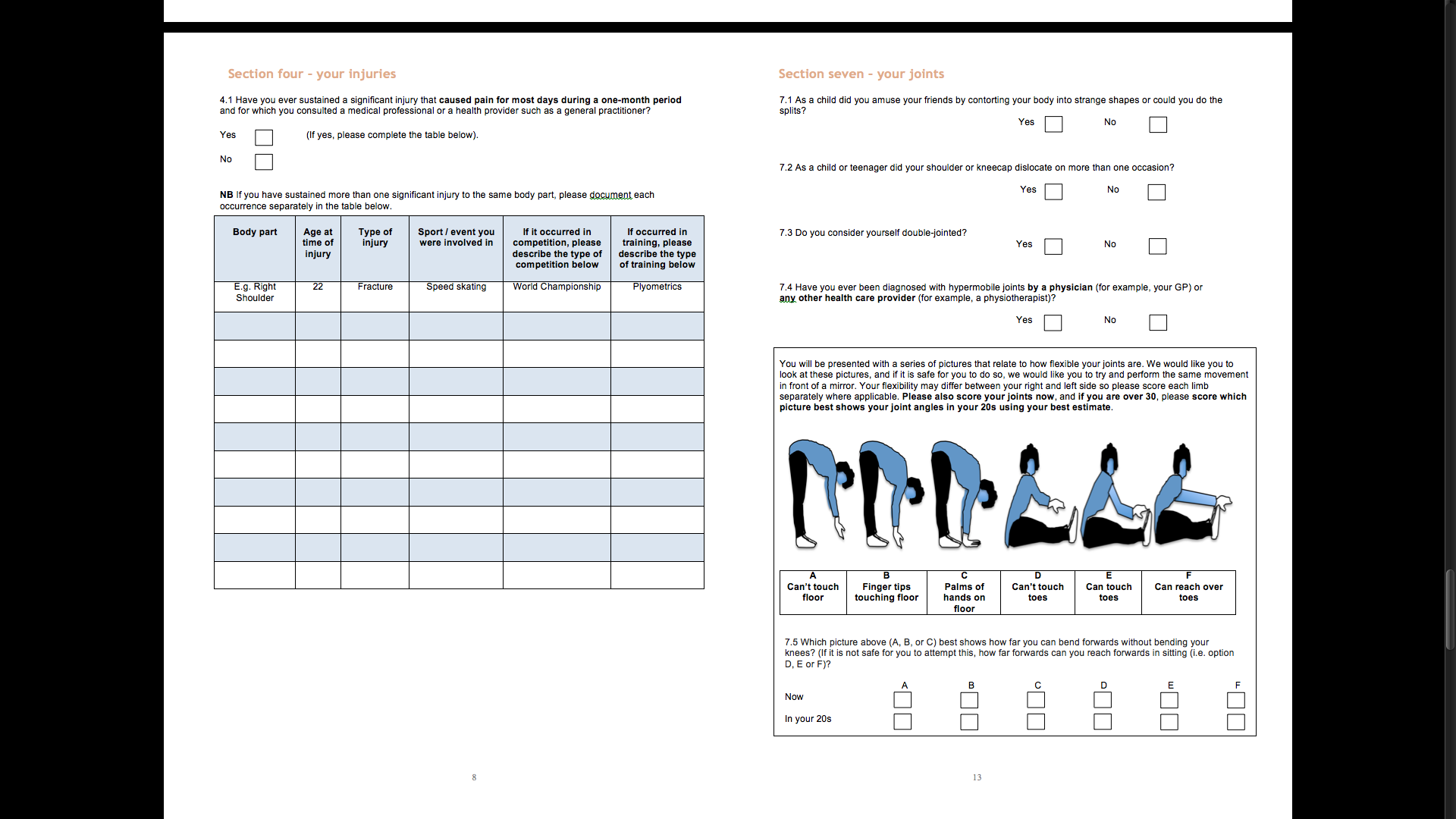


| Currently |  |  |  |  |  |  |  |  |  |  |  |  |  |  |  |  |  |
| --- | --- | --- | --- | --- | --- | --- | --- | --- | --- | --- | --- | --- | --- | --- | --- | --- | --- |
|  |  |  |  |  |  |  |  |  |  |  |  |  |  |  |  |  |  |
| 20s |  |  |  |  |  |  |  |  |  |  |  |  |  |  |  |  |  |

**1.31** Which picture below best shows how far you can **bend your elbow backwards** whilst keeping the palm of your hand facing upwards towards the ceiling? Please score for both elbows **now**, and how you were in your **20s** (if you are over 30 years of age).


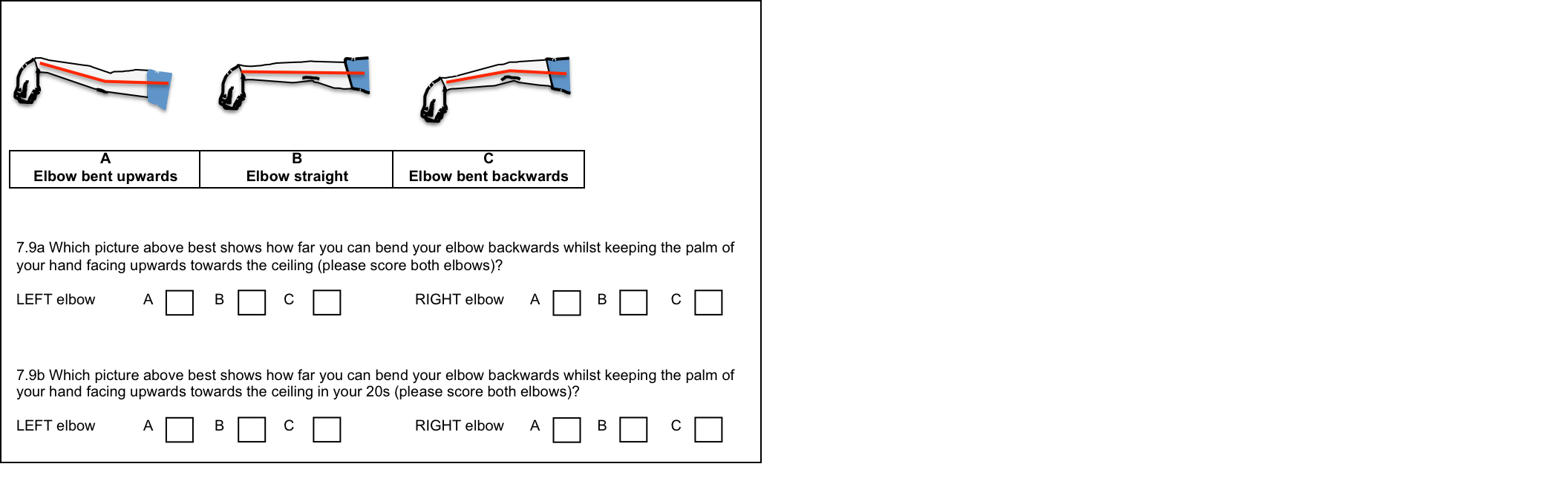


| Currently | left |  | right |  | left |  | right |  |  | left |  | right |  |  |
| --- | --- | --- | --- | --- | --- | --- | --- | --- | --- | --- | --- | --- | --- | --- |
|  |  |  |  |  |  |  |  |  |  |  |  |  |  |  |
| 20s | left |  | right |  | left |  | right |  |  | left |  | right |  |  |

**1.32** Which picture below best shows how far you can **bend your thumb to touch your wrist**? Please score both hands **now**, and in your **20s** (if you are over 30 years of age).


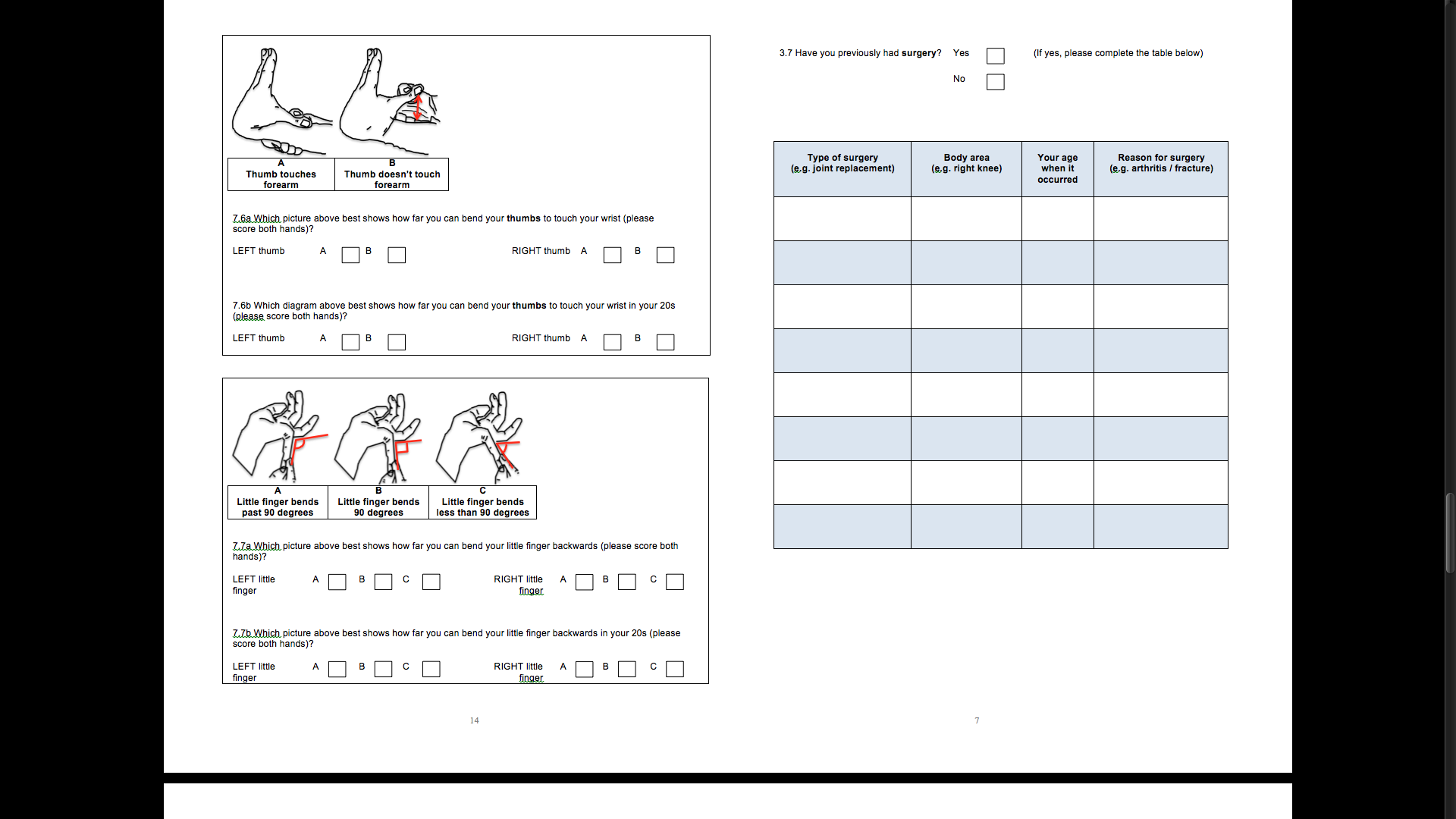


| Currently | left |  | right |  |  | left |  | right |  |  |
| --- | --- | --- | --- | --- | --- | --- | --- | --- | --- | --- |
|  |  |  |  |  |  |  |  |  |  |  |
| 20s | left |  | right |  |  | left |  | right |  |  |

**1.33** Which picture below best shows how far you can **bend your little finger backwards**. Please score both hands **now**, and in your **20s** (if you are over 30 years of age).


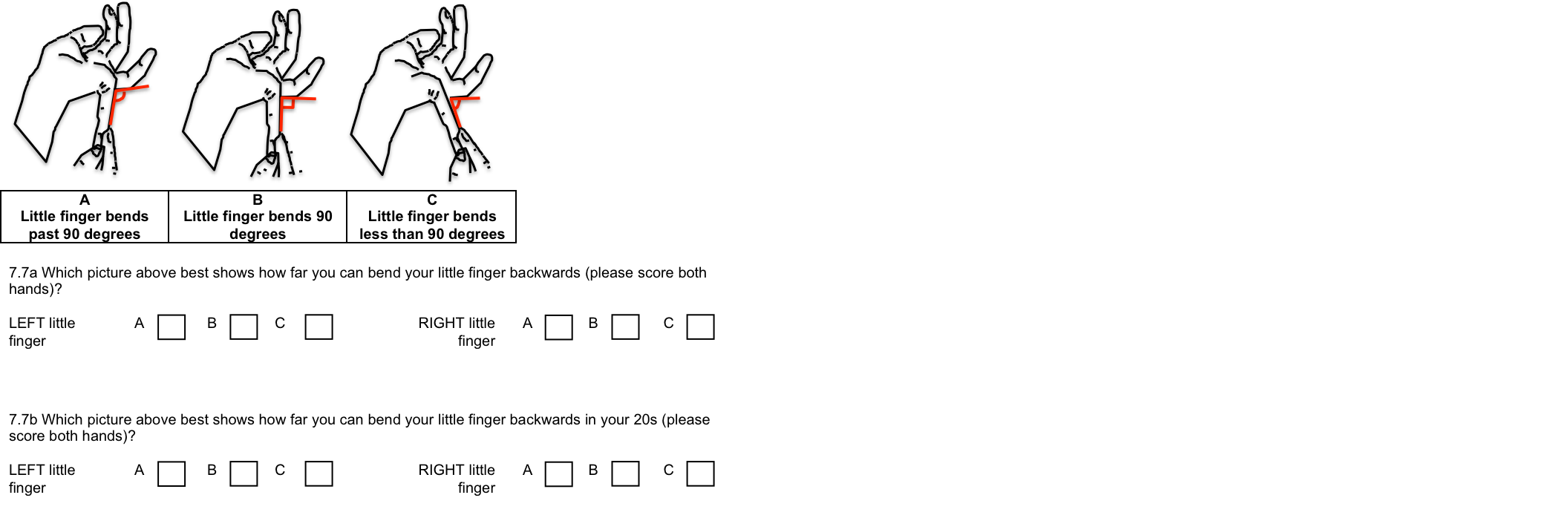


| Currently | left |  | right |  | left |  | right |  |  | left |  | right |  |  |
| --- | --- | --- | --- | --- | --- | --- | --- | --- | --- | --- | --- | --- | --- | --- |
|  |  |  |  |  |  |  |  |  |  |  |  |  |  |  |
| 20s | left |  | right |  | left |  | right |  |  | left |  | right |  |  |

**1.34** As a child did you amuse your friends by contorting your body in to strange shapes or could you do the splits?

| Yes |  | No |  |
| --- | --- | --- | --- |

**1.35** As a child or teenager did your shoulder or kneecap **dislocate** on more than one occasion?

| Yes |  | No |  |
| --- | --- | --- | --- |

**1.36** Do you consider yourself **double jointed**?

| Yes |  | No |  |
| --- | --- | --- | --- |

**Section two: your ankle injury**

**Please provide as much information as possible about your current ankle injury.**

**2.1** What **date and approx. time** did you **injure** your ankle? __________

**2.2** What date did you go to **A&E**? __________

| Yes |  | No |  |
| --- | --- | --- | --- |

**2.3** Did you see a **medical** practitioner?

**2.4** Which ankle **(left/right)** did you injure? __________

**2.5 Where** did you injure your ankle?

| Home | At work | During sport/exercise | During other leisure time | Other |
| --- | --- | --- | --- | --- |

If you injured your ankle during sport, which sport was it?______________________

If during sport, was it a competition/match, or training *(please specify)____________*

**2.6** What was the **cause of injury**? *(please choose up to 3 that apply)*

| Overuse (gradual/sudden onset) |  | Non-contact trauma |  | Recurrence of previous injury |  | Contact with a person |  |
| --- | --- | --- | --- | --- | --- | --- | --- |
|  |  |  |  |  |  |  |  |
| Contact with a static object |  | Contact with moving object |  | Ground conditions |  | Weather conditions |  |
|  |  |  |  |  |  |  |  |
|  |  |  |  |  |  |  |  |
| Equipment failure |  | Footwear |  | Other:_________ |  |  |  |
|  |  |  |  |  |  |  |  |

**2.7** Please denote any **additional factors** that may have contributed to your injury:

| Slip |  | Alcohol |  | Road traffic accident |  |
| --- | --- | --- | --- | --- | --- |

**2.8** Please indicate which of the following you were wearing when you **injured** your ankle?

| Sports shoes |  | Flat shoes |  | Heels |  | High top |  | Boots |  | Bare foot |  |
| --- | --- | --- | --- | --- | --- | --- | --- | --- | --- | --- | --- |

**2.9** Please indicate your **everyday footwear** *(i.e, wear most days*):

| Sports shoes |  | Flat shoes |  | Heels |  | High top |  | Boots |  |
| --- | --- | --- | --- | --- | --- | --- | --- | --- | --- |

**2.10** Please indicate if any of the following have occurred, and for how long, OR if you anticipate the following happening.

|  | Need(ed) crutches |  | Miss(ed) work |  | Miss(ed) sport |  | Take(n) pain killers |  |
| --- | --- | --- | --- | --- | --- | --- | --- | --- |
| If known, for how many days (d) or weeks (w) |  | |  | |  | |  | |

**2.11** As a **result** of your injury have you experienced any of the following?

| Swelling |  | Bruising |  | Stiffness |  | Looseness (instability) |  |
| --- | --- | --- | --- | --- | --- | --- | --- |

**2.12** Please indicate your **level of pain** on a scale between ‘0’ no pain and ‘10’ the worst pain imaginable? **At the time of injury** _____ and your current **level of pain now** _____

**2.13** Please indicate what **further treatment** you have been told you will need after A&E and what (if any) referrals to another specialist you have been given. *Please tick all that apply below:*

| Put in a cast |  | Referral to GP |  | Operation |  | Referral to NHS physiotherapy |  | |  |
| --- | --- | --- | --- | --- | --- | --- | --- | --- | --- |
|  |  |  |  |  |  |  |  | |  |
| Fracture clinic |  | Nurse |  | 2^nd^ x-ray |  | No further treatment or referral |  | |  |
|  |  |  |  |  |  |  |  | |  |
| Consultant |  | Private paid physiotherapy |  | MRI |  |  |  | |  |
|  | | |  |  |  |  | |  | |
| Other (please specify) __________________ | | |  |  |  |  | |  | |

**2.14 Not including your current** injury, please tell us about any previous significant **ankle** injuries that you have had *(significant = any ankle injury that affected you for most days for at least one week, affected = affected your normal day to day activities).*

| **Side (left/right)** | **Age** | **How long did the injury affect you?** | **Types of treatment you received for this injury** | | | | | | | |  |
| --- | --- | --- | --- | --- | --- | --- | --- | --- | --- | --- | --- |
|  |  |  |  |  |  |  |  |  |  |  |  |
|  |  |  | Put in cast |  | Surgery |  | NHS physio |  | Saw GP |  |  |
|  |  |  |  |  |  |  |  |  |  |  |  |
|  |  |  | Private paid physio |  | other |  | No treatment | | |  |  |
|  |  |  |  |  |  |  |  |  |  |  |  |
|  |  |  |  |  |  |  |  |  |  |  |  |
|  |  |  | Put in cast |  | Surgery |  | NHS physio |  | Saw GP |  |  |
|  |  |  |  |  |  |  |  |  |  |  |  |
|  |  |  | Private paid physio |  | other |  | No treatment | | |  |  |
|  |  |  |  |  |  |  |  |  |  |  |  |
|  |  |  |  |  |  |  |  |  |  |  |  |
|  |  |  | Put in cast |  | Surgery |  | NHS physio |  | Saw GP |  |  |
|  |  |  |  |  |  |  |  |  |  |  |  |
|  |  |  | Private paid physio |  | other |  | No treatment | | |  |  |
|  |  |  |  |  |  |  |  |  |  |  |  |

**2.15** Please tell us about any other **significant injuries** that you have had, including the **body location** and how long you were **affected** *(significant = you were affected for most days for at least one week, affected = affected your normal day to day activities)*

| **Body location** | **Side (left/right)** | **Age** | **How long did the injury affect you?** | **Types of treatment you received for this injury** | | | | | | | |
| --- | --- | --- | --- | --- | --- | --- | --- | --- | --- | --- | --- |
|  |  |  |  |  |  |  |  |  |  |  |  |
|  |  |  |  | Put in cast |  | Surgery |  | NHS physio |  |  |  |
|  |  |  |  |  |  |  |  |  |  |  |  |
|  |  |  |  | Private paid physio |  | Saw GP |  | Other |  |  |  |
|  |  |  |  |  |  |  |  |  |  |  |  |
|  |  |  |  | No treatment |  |  |  |  |  |  |  |
|  |  |  |  |  |  |  |  |  |  |  |  |
|  |  |  |  |  |  |  |  |  |  |  |  |
|  |  |  |  | Put in cast |  | Surgery |  | NHS physio |  |  |  |
|  |  |  |  |  |  |  |  |  |  |  |  |
|  |  |  |  | Private paid physio |  | Saw GP |  | Other |  |  |  |
|  |  |  |  |  |  |  |  |  |  |  |  |
|  |  |  |  | No treatment |  |  |  |  |  |  |  |
|  |  |  |  |  |  |  |  |  |  |  |  |
|  |  |  |  |  |  |  |  |  |  |  |  |
|  |  |  |  | Put in cast |  | Surgery |  | NHS physio |  |  |  |
|  |  |  |  |  |  |  |  |  |  |  |  |
|  |  |  |  | Private paid physio |  | Saw GP |  | Other |  |  |  |
|  |  |  |  |  |  |  |  |  |  |  |  |
|  |  |  |  | No treatment |  |  |  |  |  |  |  |
|  |  |  |  |  |  |  |  |  |  |  |  |

**Section three: ankle function assessment prior to this injury**

For the whole of this next section thinking about the ankle you have just injured, we want to know what that ankle function was like **before** you hurt it. Please answer all the following questions relating to **how your ankle functioned PRIOR to your current injury.**

| **3.1** **Prior** to your injury did your ankle **ever** feel ‘**loose**’? | | | | | | | | |  |  | | |  | |  |  |
| --- | --- | --- | --- | --- | --- | --- | --- | --- | --- | --- | --- | --- | --- | --- | --- | --- |
| Never |  | Rarely |  | Sometimes |  | Often |  | Always | | |  |  |  |  |  |  |
| **3.2** **Prior** to your injury did your ankle **ever** feel like it was going to ‘**give way**’? | | | | | | | | | | | | | |  | |  |
| Never |  | Rarely |  | Sometimes |  | Often |  | Always | | |  |  |  |  |  |  |
| **3.3 Prior** to your injury did your ankle **ever actually ‘give way’**? | | | | | | | | | | | | | |  | |  |
| Never |  | Rarely |  | Sometimes |  | Often |  | Always | | |  |  |  |  |  |  |
| **3.4 Prior** to your current injury, have you ever been diagnosed with Chronic Ankle Instability (CAI)? | | | | | | | | | | | | | | | | |
| Yes |  | No |  |  |  |  |  |  | | |  |  |  |  |  |  |
| If yes, who diagnosed CAI? | | | | | | | | | | | | | |  | |  |

| Doctor |  | Nurse |  | Physio |  | Consultant |  | Other__________ |
| --- | --- | --- | --- | --- | --- | --- | --- | --- |

| What year were you diagnosed with CAI? __________ |
| --- |

**Foot and ankle outcome score (FAOS)** This survey asks for your view about **your foot/ankle prior to your injury**. This information will help us keep track of how you feel about your foot/ankle and how well you were able to do your usual activities. Answer every question by ticking the appropriate box, only one box for each question. If you are unsure about how to answer a question, please give the best answer you can.

**3.5 Symptoms**

These questions should be answered thinking of the foot/ankle symptoms **during the last week prior to injury.**

| a. Do you have **swelling** in your foot/ankle? | | | | | | |  |  | | |  |  | | |  |  |  |
| --- | --- | --- | --- | --- | --- | --- | --- | --- | --- | --- | --- | --- | --- | --- | --- | --- | --- |
| Never |  | Rarely |  | Sometimes |  | Often | | |  | Always | | |  |  |  |  |  |
| b. Do you feel **grinding, hear clicking** or any other type of **noise** when your foot/ankle moves? | | | | | | | | | | | | | | | | | |
| Never |  | Rarely |  | Sometimes |  | Often | | |  | Always | | |  |  |  |  |  |
| c. Does your foot/ankle **catch** or hang up when moving? | | | | | | | | | | | | | | | | |  |
| Never |  | Rarely |  | Sometimes |  | Often | | |  | Always | | |  |  |  |  |  |
| d. Can you **straighten** your foot/ankle fully? | | | | | | | | | | | | | | | | |  |
| Always |  | Often |  | Sometimes |  | Rarely | | |  | Never | | |  |  |  |  |  |
| e. Can you **bend** your foot/ankle fully? | | | | | | | | | | | | | | | | |  |
| Always |  | Often |  | Sometimes |  | Rarely | | |  | Never | | |  |  |  |  |  |

**3.6 Stiffness**

The following questions concern the amount of joint stiffness you have experienced during the **last week prior to your injury** in your foot/ankle. Stiffness is a sensation of restriction or slowness in the ease with which you move your joints.

| a. How severe is your foot/ankle **stiffness** after first wakening in the morning? | | | | | | | | | | |
| --- | --- | --- | --- | --- | --- | --- | --- | --- | --- | --- |
| None |  | Mild |  | Moderate |  | Severe |  | Extreme |  |  |
| b. How severe is your foot/ankle stiffness after sitting, lying or resting **later in the day**? | | | | | | | | | | |
| None |  | Mild |  | Moderate |  | Severe |  | Extreme |  |  |

**3.7 Pain**

| a. How often do you experience foot/ankle **pain**? | | | | | | | | | | |  |
| --- | --- | --- | --- | --- | --- | --- | --- | --- | --- | --- | --- |
| Never |  | Monthly |  | Weekly |  | Daily |  | Always |  |  |  |

**3.8** What amount of foot/ankle pain have you experienced during the **last week prior to your injury** during the following activities?

| a. Twisting/pivoting on your foot/ankle | | | | | | | | | | |
| --- | --- | --- | --- | --- | --- | --- | --- | --- | --- | --- |
| None |  | Mild |  | Moderate |  | Severe |  | Extreme |  |  |
| b. Straightening foot/ankle fully | | | | | | | | | | |
| None |  | Mild |  | Moderate |  | Severe |  | Extreme |  |  |
| c. Bending foot/ankle fully | | | | | | | | | | |
| None |  | Mild |  | Moderate |  | Severe |  | Extreme |  |  |
| d. Walking on flat surface | | | | | | | | | | |
| None |  | Mild |  | Moderate |  | Severe |  | Extreme |  |  |
| e. Going up or down stairs | | | | | | | | | | |
| None |  | Mild |  | Moderate |  | Severe |  | Extreme |  |  |
| f. At night while in bed | | | | | | | | | | |
| None |  | Mild |  | Moderate |  | Severe |  | Extreme |  |  |
| g. Sitting or lying | | | | | | | | | | |
| None |  | Mild |  | Moderate |  | Severe |  | Extreme |  |  |
| h. Standing upright | | | | | | | | | | |
| None |  | Mild |  | Moderate |  | Severe |  | Extreme |  |  |

**3.9** The following questions concern your physical function. By this we mean your ability to move around and to look after yourself. For each of the following activities please indicate the degree of difficulty you have experienced in the **last week prior to your injury** due to your foot/ankle.

| a. Descending stairs | | | | | | | | | | |
| --- | --- | --- | --- | --- | --- | --- | --- | --- | --- | --- |
| None |  | Mild |  | Moderate |  | Severe |  | Extreme |  |  |
| b. Ascending stairs | | | | | | | | | | |
| None |  | Mild |  | Moderate |  | Severe |  | Extreme |  |  |

**3.10** For each of the following activities please indicate the **degree of difficulty** you have experienced during the **last week prior to your injury** due to your foot/ankle.

| a. Rising from sitting | | | | | | | | | | |
| --- | --- | --- | --- | --- | --- | --- | --- | --- | --- | --- |
| None |  | Mild |  | Moderate |  | Severe |  | Extreme |  |  |
| b. Standing | | | | | | | | | | |
| None |  | Mild |  | Moderate |  | Severe |  | Extreme |  |  |
| c. Bending to floor/pick up an object | | | | | | | | | | |
| None |  | Mild |  | Moderate |  | Severe |  | Extreme |  |  |
| d. Walking on a flat surface | | | | | | | | | | |
| None |  | Mild |  | Moderate |  | Severe |  | Extreme |  |  |
| e. Getting in/out of car | | | | | | | | | | |
| None |  | Mild |  | Moderate |  | Severe |  | Extreme |  |  |
| f. Going shopping | | | | | | | | | | |
| None |  | Mild |  | Moderate |  | Severe |  | Extreme |  |  |
| g. Putting on socks/stockings | | | | | | | | | | |
| None |  | Mild |  | Moderate |  | Severe |  | Extreme |  |  |
| h. Rising from bed | | | | | | | | | | |
| None |  | Mild |  | Moderate |  | Severe |  | Extreme |  |  |
| i. Taking off socks/stockings | | | | | | | | | | |
| None |  | Mild |  | Moderate |  | Severe |  | Extreme |  |  |
| j. Lying in bed (turning over, maintaining foot/ankle position) | | | | | | | | | | |
| None |  | Mild |  | Moderate |  | Severe |  | Extreme |  |  |
| k. Getting in/out of the bath | | | | | | | | | | |
| None |  | Mild |  | Moderate |  | Severe |  | Extreme |  |  |
| l. Sitting | | | | | | | | | | |
| None |  | Mild |  | Moderate |  | Severe |  | Extreme |  |  |
| m. Getting on/off the toilet | | | | | | | | | | |
| None |  | Mild |  | Moderate |  | Severe |  | Extreme |  |  |

**3.11** For each of the following activities please indicate the degree of difficulty you have experienced in the **last week prior to your injury** due to your foot/ankle.

| a. Heavy domestic duties (moving heavy boxes, scrubbing floors, etc) | | | | | | | | | | |
| --- | --- | --- | --- | --- | --- | --- | --- | --- | --- | --- |
| None |  | Mild |  | Moderate |  | Severe |  | Extreme |  |  |
| b. Light domestic duties(cooking, dusting etc) | | | | | | | | | | |
| None |  | Mild |  | Moderate |  | Severe |  | Extreme |  |  |

**3.12** The following questions concern your physical function when being active on a higher level. The questions should be answered thinking of what degree of difficulty you have experienced during the **last week prior to your injury** due to your foot/ankle.

| a. Squatting | | | | | | | | | | |
| --- | --- | --- | --- | --- | --- | --- | --- | --- | --- | --- |
| None |  | Mild |  | Moderate |  | Severe |  | Extreme |  |  |
| b. Running | | | | | | | | | | |
| None |  | Mild |  | Moderate |  | Severe |  | Extreme |  |  |
| c. Jumping | | | | | | | | | | |
| None |  | Mild |  | Moderate |  | Severe |  | Extreme |  |  |
| d. Twisting/pivoting on injured foot/ankle | | | | | | | | | | |
| None |  | Mild |  | Moderate |  | Severe |  | Extreme |  |  |
| e. Kneeling | | | | | | | | | | |
| None |  | Mild |  | Moderate |  | Severe |  | Extreme |  |  |

**3.13** **Quality of life (prior to injury)**

| a. How often are you aware of your foot/ankle problem? | | | | | | | | | | |
| --- | --- | --- | --- | --- | --- | --- | --- | --- | --- | --- |
| Never |  | Monthly |  | Weekly |  | Daily |  | Constantly |  |  |
| b. Have you modified your life style to avoid potentially damaging activities to your foot/ankle? | | | | | | | | | | |
| Not at all |  | Mildly |  | Moderately |  | Severely |  | Totally |  |  |
| c. How much are you troubled with lack of confidence in your foot/ankle? | | | | | | | | | | |
| Not at all |  | Mildly |  | Moderately |  | Severely |  | Extremely |  |  |
| d. In general, how much difficulty do you have with your foot/ankle? | | | | | | | | | | |
| None |  | Mild |  | Moderate |  | Severe |  | Extreme |  |  |

**Section four: health and quality of life**

**4.1** Please tell us about the type and amount of **physical activity** involved in your **work** by selecting one of the statements below:

|  |  | I am not in employment (e.g. retired, retired for health reasons, unemployed, full-time carer etc) |
| --- | --- | --- |
|  |  |  |
|  |  | I spend most of my time at work sitting (such as in an office) |
|  |  |  |
|  |  | I spend most of my time at work standing or walking. However, my work does not require much intense physical effort (e.g. shop assistant, hairdresser, security guard, child-minder etc) |
|  |  |  |
|  |  | My work involves definite physical effort including handling of heavy objects and use of tools (e.g. plumber, electrician, carpenter, cleaner, nurse, gardener, postal delivery workers etc) |
|  |  |  |
|  |  | My work involves vigorous physical activity including handling of very heavy objects (e.g. scaffolder, construction worker, refuse collector etc) |

**4.2** How many **hours** do you spend watching **television** on average a **week**?_____

**4.3** How many **hours** do you spend on a **computer** (including work, study, gaming and leisure) on average a **week**? **_____**

**4.4** Please include **all** different types of **general regular physical activity** that you do, e.g. walking (you should include walking to work, shopping, for pleasure etc), commuting by bike, and other activities around the house.

| **Type of activity:** | **Walking (all types)** | **Gardening/DIY** | **Housework/ childcare** | **Commute by bike** |
| --- | --- | --- | --- | --- |
| On average how many hours do you do each activity per week |  |  |  |  |

**4.5** How would you describe your usual walking pace?

| Slow (i.e less than 3mph) |  | Steady average |  | Brisk |  | Fast (i.e, over 4mph) |  |
| --- | --- | --- | --- | --- | --- | --- | --- |

**4.6** Please indicate all different types of **sport or leisure activities** that you do (e.g. jogging/football/gym/golf/aerobics/swimming etc), for how long each week and at what level? Please also include any **significant sport and leisure activities** that you used to do regularly.

| **Type of activity** | **Level** –  -*Leisure*  *-Personal fitness*  *-Competitive (local,national or international)* | **Intensity**  **-***Light*  *-Moderate*  *-Vigorous* | **How many hours per week** | **How many years have you done this activity?** | **Are you still doing this activity (pre current injury?)**  **-***Yes/No?* | **If not currently, how long ago did you do this activity?** |
| --- | --- | --- | --- | --- | --- | --- |
|  |  |  |  |  |  |  |
|  |  |  |  |  |  |  |
|  |  |  |  |  |  |  |
|  |  |  |  |  |  |  |

| **4.7** Are you a: | Smoker |  | Ex-smoker |  | Non-smoker |  |
| --- | --- | --- | --- | --- | --- | --- |

If you are a current/ex-smoker how many years have you smoked? _____________

How many cigarettes did you/do you smoke a day? _______

**4.8** Do you drink alcohol?

| Yes |  | No |  | Given up |  |
| --- | --- | --- | --- | --- | --- |

**4.9** How many **units** do you drink a **week**? *(1 pint of beer equates to 2.5 units of alcohol & 1 bottle of beer 1.7 units; a glass of wine equates to 2 units)________*

**4.10** Do **you currently** suffer from any of the following diseases? *Please tick all that apply*

| Rheumatoid Arthritis |  | Osteoarthritis |  | Diabetes |  | Asthma |  | Epilepsy |  |
| --- | --- | --- | --- | --- | --- | --- | --- | --- | --- |
|  |  |  |  |  |  |  |  |  |  |
| High blood pressure |  | Osteoporosis |  | Depression |  | Heart problems |  | Gout |  |

**4.11** Do **you** have a history of **falls**? (i.e., have you had more than 3 falls in a year?)

| Yes |  | No |  |
| --- | --- | --- | --- |

**4.12** Do you use **regular medication** for any of the following?

| Rheumatoid Arthritis |  | Osteoarthritis |  | Diabetes |  | Asthma |  | Epilepsy |  |
| --- | --- | --- | --- | --- | --- | --- | --- | --- | --- |
|  |  |  |  |  |  |  |  |  |  |
| High blood pressure |  | Osteoporosis |  | Depression |  | Heart problems |  | Gout |  |

**4.13** Do you **regularly** take any of the following?

| HRT medication |  | Pain killing medication |  | Oral contraceptive |  |
| --- | --- | --- | --- | --- | --- |

**4.14** if you regularly take pain killers please specify the type (e.g., paracetamol, ibuprofen etc) _____________

**4.15** If not now, have you previously regularly used an oral contraceptive?

| Yes |  | Total years? |  |
| --- | --- | --- | --- |

**4.16** Please indicate if any of **your family** suffers from any of the following diseases/ problems:

| Rheumatoid Arthritis |  | Osteoarthritis |  | Diabetes |  | Asthma |  | Epilepsy |  |
| --- | --- | --- | --- | --- | --- | --- | --- | --- | --- |
|  |  |  |  |  |  |  |  |  |  |
| High blood pressure |  | Osteoporosis |  | Depression |  | Heart problems |  | Gout |  |

**4.17** **EQ-5D-5L Health Questionnaire**

| Under each heading, please tick the ONE box that best describes your health TODAY. | |
| --- | --- |
| MOBILITY |  |
| I have no problems in walking about | ❑ |
| I have slight problems in walking about | ❑ |
| I have moderate problems in walking about | ❑ |
| I have severe problems in walking about | ❑ |
| I am unable to walk about | ❑ |
| SELF-CARE |  |
| I have no problems washing or dressing myself | ❑ |
| I have slight problems washing or dressing myself | ❑ |
| I have moderate problems washing or dressing myself | ❑ |
| I have severe problems washing or dressing myself | ❑ |
| I am unable to wash or dress myself | ❑ |
| USUAL ACTIVITIES *(e.g. work, study, housework, family or leisure activities)* |  |
| I have no problems doing my usual activities | ❑ |
| I have slight problems doing my usual activities | ❑ |
| I have moderate problems doing my usual activities | ❑ |
| I have severe problems doing my usual activities | ❑ |
| I am unable to do my usual activities | ❑ |
| PAIN / DISCOMFORT |  |
| I have no pain or discomfort | ❑ |
| I have slight pain or discomfort | ❑ |
| I have moderate pain or discomfort | ❑ |
| I have severe pain or discomfort | ❑ |
| I have extreme pain or discomfort | ❑ |
| ANXIETY / DEPRESSION |  |
| I am not anxious or depressed | ❑ |
| I am slightly anxious or depressed | ❑ |
| I am moderately anxious or depressed | ❑ |
| I am severely anxious or depressed | ❑ |
| I am extremely anxious or depressed | ❑ |

*UK (English) © 2009 EuroQol Group EQ-5D™ is a trade mark of the EuroQol Group*

The best health you can imagine

| We would like to know how good or bad your health is TODAY.  10  0  20  30  40  50  60  80  70  90  100  5  15  25  35  45  55  75  65  85  95 |
| --- |
| This scale is numbered from 0 to 100. |
| 100 means the best health you can imagine. 0 means the worst health you can imagine. |
| Mark an X on the scale to indicate how your health is TODAY. |
| Now, please write the number you marked on the scale in the box below. |

YOUR HEALTH TODAY =

The worst health you can imagine

*UK (English) © 2009 EuroQol Group EQ-5D™ is a trade mark of the EuroQol Group*

**WE WOULD LIKE TO EXTEND A HUGE THANK YOU FOR TAKING THE TIME TO COMPLETE THIS SIGNIFICANT ANKLE LIGAMENT INJURY SALI QUESTIONNAIRE.**

The information that you have provided as a member of the SALI cohort study will be invaluable to the Arthritis Research UK work into the prevention of ankle injuries, and ankle osteoarthritis.

**UPDATES**: If you would like to receive regular (annual) updates on the progress of the SALI cohort study please indicate in the box.

**PRIZE DRAW**: If you would be interested in being included in the SALI cohort study prize draw please indicate in the box. In the prize draw one study participant will be selected at random and will receive two tickets to a major UK sporting event. Prize draws will be completed at every questionnaire time point i.e. you will be entered each time you complete a SALI cohort questionnaire.

**FURTHER STUDIES**: You may be eligible to participate in ankle injury related research in the future. If you are interested, please could you tick the relevant box below. Receiving further information would not commit you to being in further studies.

Yes, I would like to receive written information

No, I would not like to receive written information

**THANK YOU AGAIN FOR YOUR IMPORTANT CONTRIBUTION**.

The SALI research study team.
